# Editorial on Special Issue “Pharmaceutical Formulations with Antimicrobial Properties”

**DOI:** 10.3390/pharmaceutics15010137

**Published:** 2022-12-30

**Authors:** Anca N. Cadinoiu, Marcel Popa

**Affiliations:** 1 Faculty of Medical Dentistry, Apollonia University of Iasi, 700511 Iasi, Romania; 2 Academy of Romanian Scientists, Ilfov Street, 050044 Bucharest, Romania

Even though numerous studies on the systemic administration of antimicrobial drugs can be found in the literature, they still have many shortcomings related to the site-specific drug delivery, unwanted side effects and even potential toxicity. To overcome the above limitations, it is necessary to explore new approaches for the administration of active principles with antimicrobial properties.

In this Special Issue, original papers have been published that bring new perspectives in the development of novel pharmaceutical formulations for antimicrobial applications. Active principles with antimicrobial properties, such as antibiotics [1,2,3,4,5], antifungals [6,7], nutraceuticals [8], silver or ZnO nanoparticles [9,10], plant materials [11,12] and biocides [13], have been used to obtain mainly controlled drug delivery systems.

A first study, in which the Chapman University School of Pharmacy, Chapman University Irvine, USA and the Department of Ophthalmology School of Medicine, University of Pittsburgh School of Medicine, USA collaborated, was designed to evaluate the stability of vancomycin eye drops in normal saline solution, phosphate-buffered saline (PBS) and balanced salt solution (BSS), while being stored for 7, 14 and 28 days at room temperature or refrigerated [1]. The study detected no difference in the stability of vancomycin over 28 days of storage at either room temperature or refrigeration, but a difference in pH and turbidity was observed.

Two other research teams from the Interdisciplinary Excellence Centre, Faculty of Pharmacy, Institute of Pharmaceutical Technology and Regulatory Affairs, University of Szeged, Hungary and the School of Engineering, Institute for Materials and Processes, University of Edinburgh, UK studied the preformulation of poly(vinylpyrrolidone)-based nanofiber mats loaded with ciprofloxacin (CIP) for oral administration [2]. The aim of this study was to increase the water solubility and diffusion of the active pharmaceutical ingredient and to study the in vitro drug release and its kinetics. CIP-loaded nanofibers with a small, uniform fiber diameter and a smooth surface morphology were obtained by electrospinning.

Another study involving researchers from Turkey, UK and Romania presents the fabrication of bacterial cellulose (BC)/polycaprolactone (PCL) patches loaded with different antibiotics (amoxicillin (AMX), ampicillin (AMP) and kanamycin (KAN)) for transdermal delivery [3]. The obtained antibiotic-loaded patches displayed micron-scale fibers and an interfibrous pore size. In addition, the microfibers presented a porous structure, which imparted improved biocompatibility and increased drug loading capacity. Moreover, from the BC/PCL patches produced, different types of antibiotics could be successfully loaded and released, regardless of their type. The obtained antibiotic patches can meet the characteristic requirements needed in local transdermal applications with accelerated release characteristics, adjustability and versatility for dressing trials, and lower the cost of pharmaceutical industries.

A multidisciplinary research team from Hungary and Croatia managed to prepare and test several new formulations based on elastic liposomes loaded with azithromycin (AZT), which differ in surface charge (cationic, neutral and anionic) [4]. Their antibacterial potential was evaluated against the *C. trachomatis* serovar D laboratory strain and the clinical isolate *C. trachomatis* serovar F. All tested liposomes efficiently delivered AZT to HeLa 229 cells infected with the laboratory *Chlamydia* strain, showing minimum inhibitory concentrations (MICs) and minimum bactericidal concentrations (MBCs) of AZT even 4–8 times lower than those obtained with free AZT. Also, AZT-loaded liposomes were effective against the clinical *Chlamydia* strain by decreasing the MIC values by two-fold compared to free AZT.

Three Research Institutes from Măgurele, Romania and three other Departments from the Faculty of Biology, University of Bucharest, Romania developed composite materials based on bioglass/ciprofloxacin/poly(methyl methacrylate) as multifunctional thin films for hard tissue implants [5]. Briefly, a thin layer of PMMA material was deposited by matrix-assisted pulsed laser evaporation (MAPLE) onto Ti substrates. A second layer consisting of bioglass + ciprofloxacin was applied by MAPLE to the initial thin film. The results of this study demonstrated that the laser-deposited coatings are biocompatible and resistant to microbial colonization and biofilm formation.

A stable and highly skin-permeable topical delivery system for itraconazole (ITZ) was obtained and tested by a multidisciplinary research team in Korea [6]. This topical delivery system aimed to improve the solubility and skin penetration of ITZ, as well as increase the storage duration at which it remains stable. The in vivo antifungal efficacy of this new system was compared with a commercially available oral formulation. The permeabilities of the obtained ITZ formulations through the artificial membrane, human skin and human nail were also evaluated. After analyzing the permeability results, the optimal formulation was tested in vivo to demonstrate its antifungal efficacy at a dose identical to that of commercially available oral ITZ, using a BALB/c mouse model of cutaneous *Candida albicans* infection.

Another study that sought to obtain pharmaceutical formulations based on antifungal drugs was published following collaboration between researchers from Saudi Arabia and Ethiopia. Butenafine hydrochloride nano lipid carrier (NLC) formulations were obtained and optimized [7]. The optimized BF-NLCopt exhibited a nanometer size with high drug entrapment efficiency. Further, the optimized BF-NLCopt was immobilized in a topical gel system for topical application for the treatment of fungal infections. The drug release pattern of BF-NLCopt was improved compared to BF-NLCopt-gel. The results obtained in the permeability tests demonstrated a good permeability of BF-NLCopt and BF-NLCopt-gel through the tested membrane. The conclusions of this study highlighted that the butenafine nano lipid carrier gel system acts as a potential delivery system in the treatment of antifungal diseases.

As is known, modulation of the immune system refers to a certain alteration in the immune response in the form of stimulation, amplification, expression or inactivation of some stage of the immune response. A material which exhibits a modifying immune system response to a threat is an immunomodulatory and this concept currently underlies the development of functional foods. The study of a group of researchers from Romania [8] aimed to evaluate the immunomodulatory properties of two novel nutraceutical formulations, one based on curcuminoids from different natural sources and another based on probiotics (*Lactobacillus acidophilus*, *Bifidobacterium animalis*) combined with actives from *Helianthus tuberosus* and *Plantago ovate* as herbal prebiotics. The results obtained are encouraging and attest to the great potential of the obtained nutraceutical formulations for their designated purposes, recommending them for further in vivo studies.

Another group of Romanian researchers proposes a new type of full-IPN hydrogel, based on chitosan and poly(vinyl alcohol), obtained by combining two cross-linking methods: covalent and physical. In fact, the authors obtained a biocomposite consisting of silver nanoparticles dispersed in the matrix of IPN, designed to exert a synergistic antimicrobial activity through the action of both chitosan and silver [9]. The biocomposite obtained is of hydrogel type, with pores whose sizes vary between 40 and 60 µm, with swelling capacity in water that depends on different factors, among which the chemical nature of the epoxy crosslinker is important. The tests showed that the obtained materials present a high inhibitory activity against *S. aureus* and *K. pneumonia*, but low activity for *P. aeruginosa* and *E. coli*, and may be considered non-cytotoxic. The swelling behavior, the mechanical properties, and the antimicrobial activity recommend the use of this biocomposite in biomedical applications as wound or oral dressings.

The same type of matrix (with hydrogel character), but covalently cross-linked with glutaric aldehyde, was used to disperse ZnO particles in order to obtain a biocomposite proposed for the treatment of bacterial infections [10]. The increased concentration of covalent cross-linker led to increased mechanical properties, but when ZnO nanoparticles were incorporated into the hydrogels, the tensile strength and elongation at break decreased compared with the sample without metal oxide NPs. The degree of covalent crosslinking and the amount of ZnO nanoparticles incorporated influence the morphology and swelling degree of hydrogels. The biocomposite is practically non-toxic and shows significant antimicrobial activity against *S. aureus*, *E. coli*, and *K. pneumonia*.

A review discusses the possibility of using plant materials in the management of many inflammatory diseases, including periodontitis—one of the most serious oral diseases which affects tissues, being caused by pathogenic bacteria and environmental factors [11]. It is well known that a series of products extracted from plants exhibit antibacterial, anti-inflammatory and antioxidant activities, and affect the periodontium structure. The review is based on the consultation of 180 bibliographic references, most of them recent, and analyzes the use and applicability of plant materials in periodontitis. Their benefits concern the biocompatibility, the lower cost and the better safety profile.

In another paper of this issue, harmala alkaloid-rich fraction (HARF) (which is hydrophobic) was included in 2-hydroxy propyl-β-cyclodextrin to obtain a host–guest complex; by doing so, the water solubility and bioavailability of HARF was improved [12]. The complex was co-encapsulated with ascorbic acid into poly(lactide-glicolide)-based nanoparticles coated with poly(ethylene glycol), the entrapment efficiency being very high (more than 80%). The obtained polymer–drug system exhibited the highest antibacterial activity against *Staphylococcus aureus* and *Escherichia coli*, and high selective antiviral activity against the H1N1 influenza virus, with minimum host toxic effects.

The last paper of the issue concerns the preparation of ligand brush nanocapsules, based on poly(vinyl pyrrolidone), coated with SiO_2_ and grafted with poly(methalic acid sodium salt) [13]. The ligand-grafted nanocapsule diameter was 888 nm, and they manifested a self-controlled antibacterial response in the pH and humidity conditions needed for medical applications. Ligand-brush NCs containing an anionic antimicrobial drug had a rapid release effect because of the repellent electrostatic force and swelling properties of the ligand brushes. The experiments proved that *Escherichia coli* was eradicated at pH 6–7 and humidity 45–100%, conditions in which most bacterial and fungal strains thrive.

The editors of this Special Issue of *Pharmaceutics*, titled *Pharmaceutical Formulations with Antimicrobial Properties*, are researchers with decades of experience in the field of controlled drug release, and they consider these articles reporting recent results concerning the obtaining of polymer–drug systems, their properties and their biomedical applications justified and useful.

The editors express their gratitude for the kindness and cooperation of the contributors, who are reputable researchers in the field and have responded to our call to contribute to this Special Issue of *Pharmaceutics* with the results of their recent research.
